# A pilot multicenter randomized controlled trial on individualized blood pressure targets versus standard care among critically ill patients with shock

**DOI:** 10.1186/s40560-025-00798-8

**Published:** 2025-05-27

**Authors:** Rakshit Panwar, Bairbre McNicholas, Ciprian Nita, Alison Gibberd, Amber-Louise Poulter, Marcia Tauares, Lauren Ferguson

**Affiliations:** 1 Intensive Care Unit, John Hunter Hospital, Newcastle, Australia; 2 School of Medicine and Public Health, University of Newcastle, Newcastle, Australia; 3 Intensive Care Unit, Galway University Hospital, Galway, Ireland; 4 University of Galway, Galway, Ireland; 5 Hunter Medical Research Institute, Newcastle, Australia; 6 University of Melbourne, Melbourne, Australia

**Keywords:** Blood pressure target, Mean arterial pressure deficit, Relative hypotension, Intensive care, Shock

## Abstract

**Background:**

Minimizing relative hypotension, or mean arterial pressure (MAP) deficit, by targeting patients’ own pre-illness MAP (individualized MAP) during vasopressor therapy is a potential strategy to improve outcomes among ICU patients with shock. We conducted a prospective, open label, parallel-group, pilot RCT to assess feasibility and safety of this intervention compared to standard care.

**Methods:**

Thirty-seven eligible patients, aged 40 years or older and receiving vasopressor support for shock, were randomly allocated to individualized MAP target (*N* = 17) or standard MAP target (*N* = 20) at two multidisciplinary ICUs in Australia and Ireland. Pre-specified endpoints were time-weighted average MAP-deficit (i.e., percentage difference between patients’ pre-illness MAP and achieved-MAP), percentage time spent with > 20% MAP-deficit, major adverse kidney events (MAKE-14), 14-day and 90-day all-cause mortality, and cardiovascular adverse events within 28 days of randomization. All comparisons of efficacy outcomes were exploratory.

**Results:**

The median MAP-deficit and percentage time with > 20% MAP-deficit with individualized MAP *vs.* standard MAP were 7% [interquartile range: 2–16] *vs.* 18% [9–23] (*p* = 0.048), and 8% [0–43] *vs.* 53% [14–75] (*p* = 0.03), respectively. MAKE-14 (2/17 (12%) *vs.* 4/20 (20%), *p* = 0.67), 14-day mortality (1/17 (6%) *vs.* 3/20 (15%), *p* = 0.61), 90-day mortality (2/17 (12%) *vs.* 4/20 (20%), *p* = 0.67) and cardiovascular adverse events were similar for both groups.

**Conclusions:**

This pilot RCT demonstrated that an individualized MAP target strategy was feasible to implement. No adverse safety signals were evident. These data and study procedures helped inform the design of a definitive RCT on the question of individualized MAP targets among critically ill patients with shock.

*Study registration*: ACTRN12618000571279.

**Supplementary Information:**

The online version contains supplementary material available at 10.1186/s40560-025-00798-8.

## Introduction

Shock is a common condition in an intensive care unit (ICU) [1, 2]. There is no good evidence to determine the most effective blood pressure (BP) for individual patients who are being managed for shock in ICU. Current recommendations on BP targets [3–5] are based on randomized controlled trials (RCTs) [6–8] that applied higher or lower BP targets without accounting for patients’ own baseline or pre-illness BP. None of the RCTs in ICU investigated a strategy of adjusting BP targets for individual patients according to their pre-illness BP.

Recently, in a multicenter prospective study, we demonstrated that nearly all ICU patients were exposed to a varying degree of time-weighted average mean arterial blood pressure (MAP) deficit, a measure of relative hypotension, during vasopressor support for shock [9]. The ‘time-weighted average MAP-deficit’ was a summary measure of percentage MAP-deficit to which patients were exposed from the time of enrollment to the last recorded MAP during vasopressor therapy in ICU. It was used to quantify the magnitude of average relative hypotension load for each patient [9]. Both the degree and the duration of such relative hypotension were independently associated with increasing risk of developing major adverse kidney events by day 14 (MAKE-14) and 14-day mortality [9]. These findings imply that a strategy of adjusting MAP targets to approximate patients’ own pre-illness MAP can substantially reduce such MAP-deficit and therefore may have a potential to improve outcomes among ICU patients with shock. By not considering patients’ pre-illness MAP, the standard MAP target of ~ 65 mmHg often results in unwarranted variation in the degree of MAP deficit that seems to be subliminally accepted in real-world practice without a sound physiological rationale.

Accordingly, with the hypothesis that targeting a patient’s own pre-illness MAP during vasopressor support will substantially minimize MAP-deficit, we conducted this pilot multicenter RCT to demonstrate feasibility, assess safety, and evaluate study procedures to inform a larger multicenter RCT.

## Materials and methods

### Study design

We conducted a prospective, open label, parallel-group, RCT at two multidisciplinary tertiary-level ICUs in Australia and Ireland. The study was prospectively registered at the Australian New Zealand clinical trial registry (ACTRN12618000571279). Approval from relevant ethics committees was obtained at each participating site HNEHREC 18/03/21/3.04 and GUH- C.A. 2491. Written informed consent was obtained from patients or their legal surrogates. If a legally authorized representative was not immediately available and the clinical context required urgent inclusion, then consent-to-continue was pursued in accordance with the ethics approval. Research coordinators at each site manually screened adult ICU patients for following eligibility criteria. The study followed the CONSORT extension for pilot and feasibility trials [10].

### Inclusion criteria

- ICU patients aged 40 years or above
- Suspected shock [9, 11], defined as clinician-initiated vasopressor therapy AND supported by any of the following within the last 24 h:
  o Lactate ≥ 2 mmol/l or base deficit ≥ 3 mmol/l
  o Urine output ≤ 0.5 ml/kg/h or < 40 ml/h for 2 or more consecutive hours
  o Respiratory rate > 22 per minute
  o Altered mentation (Glasgow Coma Score < 14).

### Exclusion criteria

- Moribund or not-for-resuscitation orders
- Receiving or in imminent need of renal replacement therapy
- Patients with an increase in serum creatinine of > 350 μmol/l from baseline
- End stage renal disease
- At least 24 h have lapsed from the time of initiation of vasopressor or inotropic support
- Patients where trauma is the main reason for the current ICU admission.
- Pregnancy, if known
- Active bleeding (clinical suspicion or ≥ 3 packed red blood cells within 24 h)
- Insufficient (less than two) pre-morbid BP readings are available
- Patients on extracorporeal support
- Potential contraindications to either higher or lower BP targets (including but not limited to)
  o Cerebral perfusion pressure guided therapy, e.g., intracranial hemorrhage or subarachnoid hemorrhage or traumatic brain injury
  o Abdominal perfusion pressure guided therapy
  o Aortic injury (dissection or postoperative)
  o Post-cardiac surgery
  o Any other condition requiring higher or lower BP target specifically in the opinion of treating team.

Patients were randomly assigned, using opaque sealed envelopes, to standard MAP target group (control) or individualized MAP target group (intervention). Randomization was done using a unique computer-generated, permuted block randomization method with random block sizes, stratified for each site.

In the standard MAP group, vasopressor support was titrated to maintain a default MAP of 65 mmHg, unless a different MAP target was specified by the treating team. This was done to ensure that the control arm closely reflected the standard real-world practice, and we did not seek to artificially modify clinicians’ standard practice in the ‘standard care arm’.

In the intervention group, individualized MAP management involved targeting a patient’s own pre-illness MAP ± 2 mmHg for the duration of vasopressor therapy for up to a maximum of 5 days. The individualized MAP target was limited to a range of 55 mmHg to 95 mmHg. In other words, if the estimated pre-illness MAP happened to be lower than 55 mmHg or higher than 95 mmHg, the MAP target would still be from within the above range. Following steps were used to estimate pre-illness MAP from a patient’s most recent pre-illness BP readings.

Step 1 Trace up to 5 (minimum of 2) BP measurements, recorded at least 12 h apart, starting with the most recent reading available from the last 3 years when the patient was deemed to be in usual health from:
  BP recorded during ambulatory BP monitoring, outpatient GP/specialist visits, pre-admission clinics or echocardiography clinics. If unavailable, then
  BP recorded on the observation charts from the last 48 h of a previous hospitalization.
Step 2 Derive pre-illness MAP as follows:
  Convert BP readings recorded in systolic BP/diastolic BP format to MAP using the equation, MAP = diastolic BP + 1/3 (systolic BP—diastolic BP)
  The mean of these available MAP values was considered as pre-illness MAP.

Treating clinicians were allowed to use their discretion in adjusting these BP targets in case any new condition developed resulting in loss of equipoise or if they were concerned about an increase in noradrenaline-equivalent dose particularly beyond the threshold of 0.75 µg/kg/minute. The protocol permitted treating clinicians to adjust the MAP targets, as clinically indicated, to next highest achievable and acceptable target. Vasopressor dose was titrated to effect. Randomization was stratified for each site and therefore both parallel groups had an equal likelihood of receiving the same batch of vasopressors at respective sites. Study intervention ceased if a patient was considered well enough by the treating clinician for discharge out of ICU or if the intervention was no longer in the patients’ best interest or if the consent was withdrawn.

There were no restrictions to concomitant therapies provided to patients in the study. Participating sites were requested to adhere to best practice guidelines, regardless of assigned BP targets, in relation to other potentially confounding co-interventions such as fluid management, blood transfusion, sedation interruption, ventilator weaning, nutrition, and use of steroids.

### Data collection

The time of initiation of a continuous vasopressor or inotrope infusion was identified as T0 for each patient enrolled during the study period. Four hourly data on MAP and norepinephrine-equivalent vasopressor dose were recorded until a patient was weaned off vasopressor support for at least 24 h or up to a maximum of 5 days from T0, whichever was earlier. Other data points included demographics, Acute Physiology and Chronic Health Evaluation (APACHE) III risk score (for the 24 h prior to randomization) [12], pre-illness blood pressure readings, diagnosis at ICU admission, type of shock, past medical comorbidities, volume of intravenous fluid administered within the 24 h prior to randomization, exposure to nephrotoxic agents within 72 h prior to randomization, pre-morbid creatinine level, and the most recent serum lactate and serum creatinine levels obtained at or just prior to T0, and the time of randomization. MAKE-14 was defined as a composite measure of death, new initiation of renal replacement therapy (RRT), or doubling of serum creatinine from the pre-morbid level at day 14 [13, 14]. The most recent pre-morbid serum creatinine level was sourced from medical records within 12 months before hospital admission or if unavailable, then from the current hospital stay at least 7 days before ICU admission. When neither of these are available, the pre-morbid serum creatinine was estimated following the Kidney Disease: Improving Global Outcome (KDIGO) guidelines [15]. Time from T0 to randomization and time from randomization to cessation of vasopressors were recorded. Peak creatinine, highest MAP and lowest MAP were also recorded for each day. Cardiovascular events such as atrial arrhythmia, ventricular arrhythmia, mesenteric or myocardial ischemia, Takotsubo cardiomyopathy, bilateral digital ischemia, cardiac arrest, and ischemic stroke were monitored for 28 days following randomization for all patients.

### Outcomes

The feasibility outcome was time-weighted average of area-under-the-curve (AUC) percentage MAP-deficit during the active treatment period. MAP-deficit at each 4-hourly time-point was calculated as the percentage deficit between the pre-illness MAP and the achieved-MAP in ICU [i.e., 100 * (pre-illness MAP—achieved MAP)/pre-illness MAP] [9, 16]. Additional explanation for this parameter, including an illustration, is provided in the supplement. Other feasibility and safety outcomes included percentage timepoints spent with > 20% MAP-deficit, percentage timepoints spent with MAP < 65 mmHg, all-cause mortality within 14 days and 90 days of randomization, time to death within 90 days after randomization, MAKE-14 [9], new-onset significant acute kidney injury (AKI) [9], need for RRT, peak rise in serum creatinine within 14 days, cardiovascular events within 28 days of randomization, ICU and hospital length of stay among survivors. All comparisons of efficacy outcomes were considered exploratory.

### Statistical analyses

Analyses were performed using a standard statistical software (R version 4.3.1) [17]. Statistical tests for differences in characteristics and feasibility and safety outcomes were conducted using the *finalfit* package in R [18]. Linear mixed-effects models were fit using the *lme4* package [19]. Based on our prior observational study [9], a sample size of 50 was required to demonstrate an absolute difference of 8% in the primary endpoint of percentage MAP-deficit between the two arms, assuming a mean of 10% MAP-deficit and a standard deviation of 9% in the standard care arm, at an alpha level of 0.05 and power of 80%. However, the study was terminated after enrollment of 37 patients as a grant was secured for the next phase RCT and therefore it became a priority to commence phase 3 RCT. Descriptive statistics were reported for clinical characteristics, process of care measures and outcomes. For categorical variables, counts and percentages were reported and P-values were from Fisher’s exact test due to small counts in this pilot study. For continuous variables, medians and interquartile ranges [IQRs] were reported and P-values were from Wilcoxon rank sum tests. A two-sided P-value of 0.05 was considered statistically significant. To compare selected continuous outcomes with repeated measures in the two study arms, linear mixed-effects models were fit, with a random intercept for each participant and fixed effects for treatment (individualized care versus standard care), days from initiation of vasopressor therapy to randomization (a linear term), time since randomization (days, a linear term), and an interaction term for treatment and time. The modeled outcomes were percentage MAP-deficit, achieved MAP, vasopressor dose, highest daily MAP, lowest daily MAP, and daily peak creatinine. Coefficients and their 95% CIs were reported for the fixed effect of treatment and the interaction term between time and treatment. Only non-missing observations during the study period were included in the analyses. Mixed-effects models are generally well-suited to handle missing data in repeated measures variables. Protocol variations implemented during the course of this pilot RCT are listed in Table S1. All analyses followed intention-to-treat principle.

## Results

**Fig. 1:**
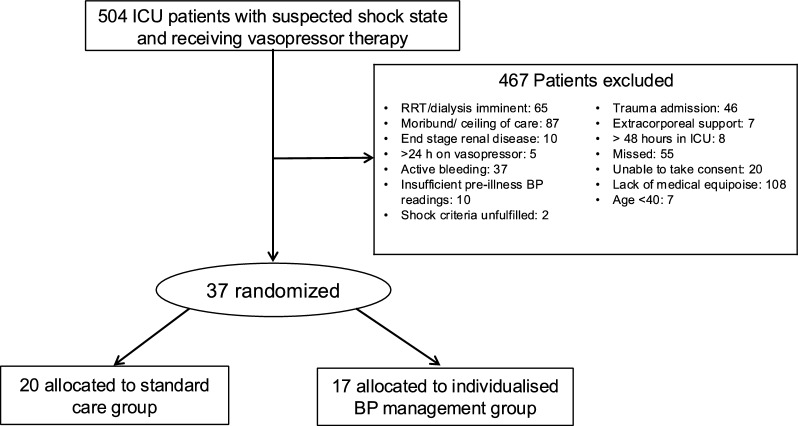
Patient flow diagram

We screened 504 patients at two multi-disciplinary ICUs in Australia and Ireland, enrolling 37 patients between December 2018 and March 2023. Of these, 20 patients were assigned to the standard MAP group and 17 patients were assigned to the individualized MAP group (Fig. 1); 22 were recruited in Australia and 15 recruited in Ireland. All enrolled patients were followed up until day 90 or the day of death whichever was earlier. Outcomes were available for all patients.

As shown in Table 1, there were no significant between-group differences regarding age, gender, APACHE III score, comorbidities, diagnosis organ system, serum lactate, serum creatinine at randomization, and the time from T0 to randomization. The amount of intravenous fluid administration and exposure to nephrotoxic agents prior to randomization were similar in between the two groups. All patients who were randomized to standard MAP group had septic shock, while four participants randomized to individualized MAP group had cardiogenic shock or a mix of septic and cardiogenic shock. The number and period of pre-illness blood pressure readings traced per patient were similar across both groups. The pre-illness MAP and the MAP at randomization were similar in between the two groups. Exposure to blood transfusion and nephrotoxic agents after randomization were also similar for both groups. Post randomization, the achieved MAP (median 77 [IQR: 75–81] *versus* 83 [IQR: 80–85], *p* = 0.05) was higher in the intervention group.

**Table 1:** Baseline characteristics and process of care variables

Characteristics	Standard MAP (*n* = 20)	Individualized MAP (*n* = 17)
Age, median [IQR], years	67 [58–78]	69 [57–74]
Males, *n* (%)	12 (60%)	9 (53%)
APACHE III score, median [IQR]	68 [53–82]	85 [62–92]
Co-morbidities, *n* (%)		
Chronic hypertension	14 (70%)	8 (47%)
Diabetes mellitus	6 (30%)	5 (29%)
Chronic obstructive pulmonary disease	5 (25%)	2 (12%)
Ischemic heart disease	1 (5%)	3 (8%)
Congestive heart failure	4 (20%)	1 (6%)
Chronic kidney disease	0 (0%)	0 (0%)
Valvular heart disease	1 (5%)	0 (0%)
Peripheral vascular disease	2 (10%)	0 (0%)
Diagnosis organ system, *n* (%)		
Gastrointestinal	7 (35%)	2 (12%)
Respiratory	4 (20%)	1 (6%)
Cardiovascular	0 (0%)	3 (18%)
Sepsis	6 (30%)	7 (41%)
Neurological	1 (5%)	1 (6%)
Other	1 (5%)	1 (6%)
Type of shock, *n* (%)		
Septic	20 (100%)	13 (76%)
Cardiogenic	0 (0%)	3 (18%)
Mixed	0 (0%)	1 (6%)
Period between pre-illness blood pressure (BP) measurements and randomization, median [IQR], weeks	10.2 [3.1–16.3]	13 [6.1–33]
Number of pre-illness BP readings per patient, n (%)		
2	3 (15)	2 (12)
3	2 (10)	3 (18)
4	7 (35)	3 (18)
5	8 (40)	9 (53)
Pre-illness mean arterial pressure, median [IQR], mmHg	97 [93–101]	94 [83–98]
MAP at randomization, median [IQR], mmHg	73 [68–79]	74 [70–77]
Serum lactate at randomization, median [IQR], mmol/l	1.4 [1.0–2.9]	2.2 [1.8–3.3]
Pre-illness serum creatinine, median [IQR], micromole/l	77 [62–90]	80 [71–93]
Serum creatinine at randomization, median [IQR], micromole/l	127 [76–168]	146 [87–185]
Intravenous fluid given within 24 h prior to randomization, median [IQR], ml	3000 [1372–3689]	2852 [1882–4589]
Exposure to nephrotoxic agents within 72 h prior to randomization, n (%)		
ACE inhibitor or angiotensin receptor blocker	10 (50%)	4 (24%)
Intravenous contrast	8 (40%)	11 (65%)
Non-steroidal anti-inflammatory drug	7 (35%)	4 (24%)
Aminoglycoside	7 (35%)	8 (47%)
Vancomycin	5 (25%)	6 (35%)
Number of nephrotoxic agents within 72 h prior to randomization, median per patient [IQR]	2 [1, 2]	2 [1–3]
Exposure to nephrotoxic agents within 14 days after randomization, n (%)		
ACE inhibitor or angiotensin receptor blocker	7 (35%)	3 (18%)
Intravenous contrast	9 (45%)	6 (35%)
Non-steroidal anti-inflammatory drug	7 (35%)	7 (41%)
Aminoglycoside	5 (25%)	5 (29%)
Vancomycin	9 (45%)	6 (35%)
Calcineurin inhibitor	0 (0%)	1 (6%)
Number of nephrotoxic agents within 14 days after randomization, median per patient [IQR]	2 [1, 2]	2 [1, 2]
Exposure to blood transfusion within 5 days after randomization, *n* (%)	6 (30%)	5 (29%)
Achieved MAP ^#^ post-randomization, median [IQR], mmHg	77 [75–81]	83 [80–85]
Percentage of time spent with MAP < 65 mmHg^**Ω**^, median [IQR], %	3 [0–13]	0 [0–4]
Time from ICU admission to randomization, median [IQR], hrs	17 [14–29]	19 [17–26]
Time from T0^§^ to randomization, mean (SD), hrs	17 [16–32]	20 [17–33]
Time from randomization to cessation of vasopressors, median [IQR], hrs	72 [29–135]	107 [49–156]
ICU length of stay post-randomization among survivors, median [IQR], days	5.4 [4.1–10.4]	8.3 [3.3–11.3]
Hospital length of stay post-randomization among survivors, median [IQR], days	18.2 [8.3- 29.1]	14.5 [10.8–28.2]

IQR: interquartile range, *APACHE* Acute Physiology and Chronic Health Evaluation, *BP* blood pressure, *MAP* mean arterial pressure, *ACE* angiotensin converting enzyme

^§^ T0- Time-point, when vasopressor or inotrope support was initiated

^#^ Achieved-MAP was derived as the time-weighted average of 4-hourly values over the active treatment period

^**Ω**^ % Time spent with MAP < 65 mmHg = [Σ(time-periods with MAP < 65 mmHg)/total time with available MAP data]*100

### Feasibility outcomes

**Fig. 2:**
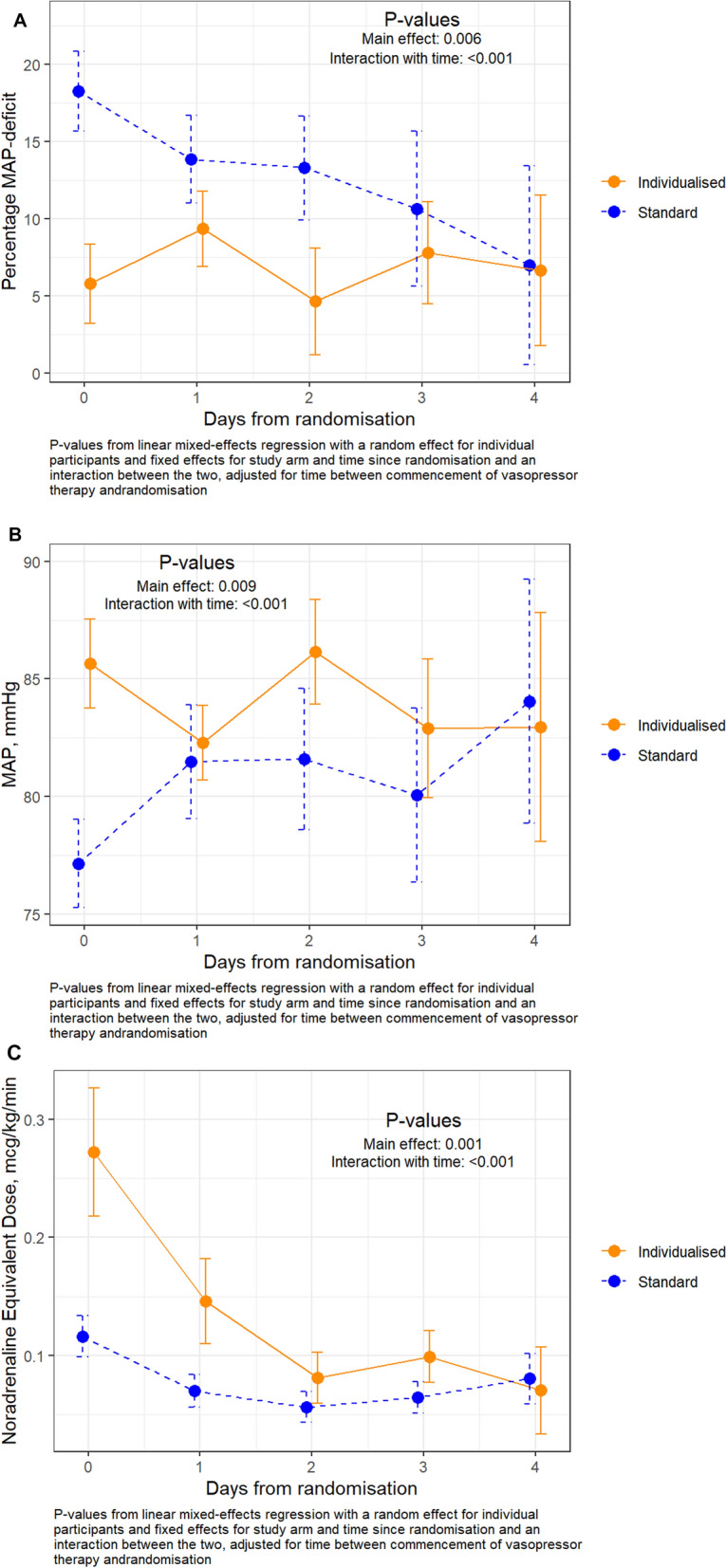
**A** Percentage MAP-deficit (daily mean and 95% confidence intervals) over time in standard MAP target group versus individualized MAP target group. **B** Achieved MAP in ICU (daily mean and 95% confidence intervals) over time in standard MAP target group versus individualized MAP target group. **C** Vasopressor dose (daily mean with 95% confidence interval) in ICU over time in standard MAP target group versus individualized map target group

**Fig. 3:**
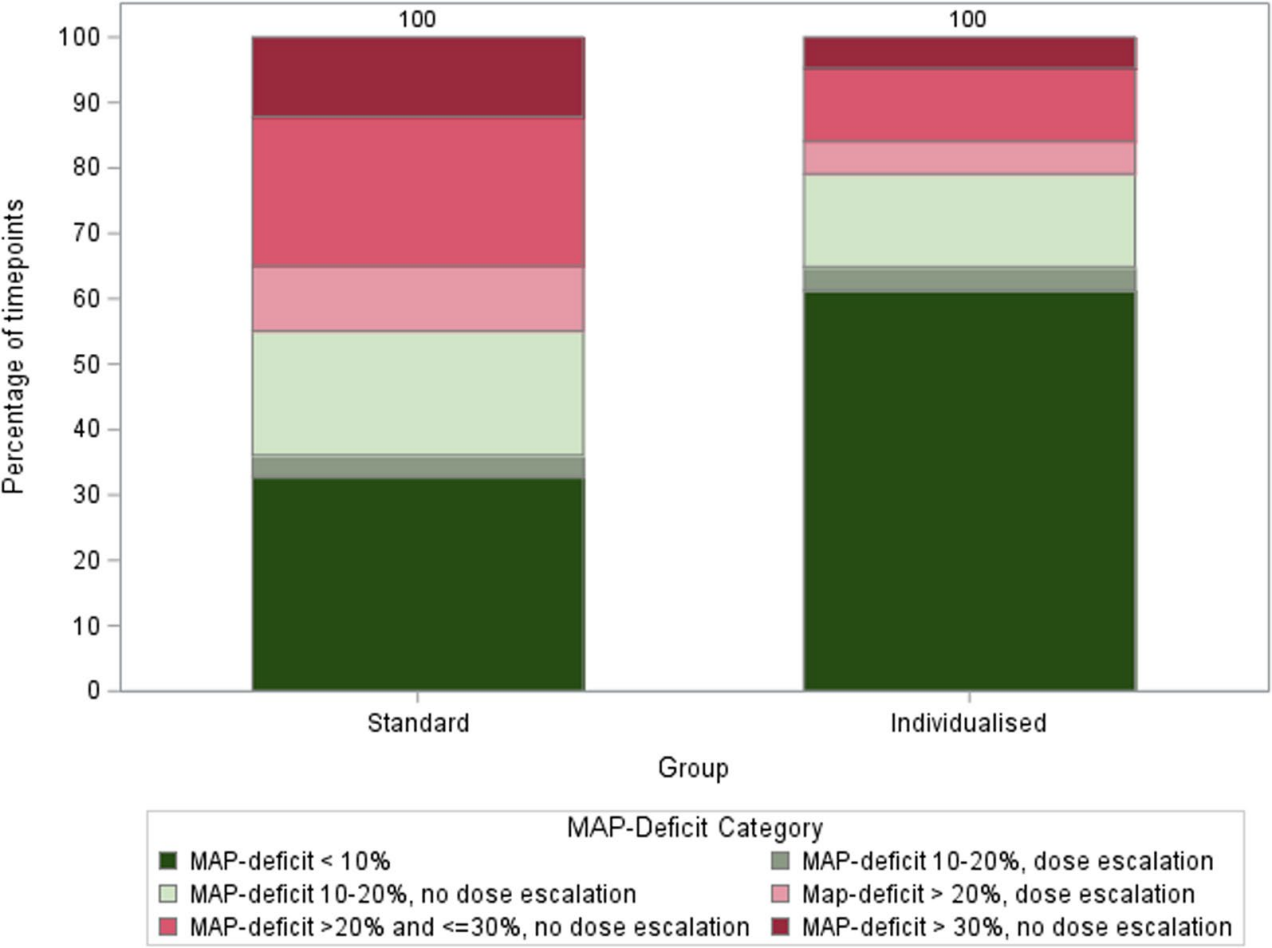
MAP-deficit profile in the two groups

As shown in Table 2, compared to the standard MAP group, patients enrolled in the individualized MAP group had lower time-weighted average MAP deficit (18% [9–23] *vs.* 7% [2–16], *p* = 0.048) and spent less percentage of time with > 20% MAP deficit (53% [IQR: 14–75] *vs.* 8% [IQR: 0–43], *p* = 0.03) during vasopressor therapy. The two groups were well separated over time in relation to the daily average MAP deficit (Fig. 2A and Figure S1) and daily average MAP achieved in ICU (Fig. 2B and Figure S2). The vasopressor dose (Fig. 2C) over the first 5 days of vasopressor therapy was higher for individualized MAP group compared to the standard MAP group. Figure 3 illustrates a clear divergence in vasopressor management strategies for relative hypotension, resulting in two distinct profiles of MAP-deficit and its management, between the two groups. As seen in the figure, the individualized MAP group spent a greater proportion of time with MAP-deficits < 10%, whereas the standard group showed a broader spread across all other deficit categories, including higher proportions in the > 20% range with or without escalation. This pattern reflects a more targeted approach in the individualized MAP group and supports the fidelity of intervention delivery. Daily doses of individual vasopressors, including norepinephrine, adrenaline, vasopressin, and dobutamine are shown in supplementary Table S2. The highest and lowest daily MAPs over the first 5 days are shown for both groups in Figures S3-S4.

**Table 2:** Feasibility and safety outcomes

	Standard MAP (*n* = 20)	Individualized MAP (*n* = 17)	*P* value
MAP-deficit^*^ (time-weighted average), median [IQR], %	18 [9–23]	7 [2–16]	0.048
Percentage of time spent with > 20% MAP-deficit^**χ**^, median [IQR], %	53 [14–75]	8 [0–43]	0.03
Day 14 all-cause mortality, *n* (%)	3 (15)	1 (6)	0.61
Major adverse kidney event^**δ**^ within 14 days of randomization, *n* (%)	4 (20)	2 (12)	0.67
Peak increase in serum creatinine^**γ**^ within 14 days, median [IQR], %	57 [33–121]	45 [14–61]	0.54
New significant AKI^**ϕ**^ within 14 days of randomization, *n* (%)	2 (10)	0 (0)	0.49
Need for renal replacement therapy within 14 days of randomization, *n* (%)	1 (5)	1 (6)	> 0.99
Cardiovascular adverse events within 28 days of randomization, *n* (%)			
Atrial arrhythmia	5 (25)	4 (24)	> 0.99
Ventricular arrhythmia	4 (20)	3 (18)	> 0.99
Mesenteric or myocardial ischemia	0 (0)	0 (0)	–
Takotsubo cardiomyopathy	0 (0)	1 (6)	0.46
Bilateral digital ischemia	0 (0)	1 (6)	0.46
Cardiac arrest	0 (0)	1 (6)	0.46
Ischemic stroke	1 (5)	0 (0)	> 0.99
Day 90 all-cause mortality, *n* (%)	4 (20)	2 (12)	0.67

*IQR* interquartile range, *MAP* mean arterial pressure, *mmHg*; *AKI* acute kidney injury

^*^ MAP-deficit = [(pre-illness MAP − achieved MAP)/pre-illness MAP]*100, using positive incremental area-under-the-curve of MAP-deficit during vasopressor therapy

^**χ**^ % Time spent with > 20% MAP-deficit = [Σ(time-periods with > 20% MAP-deficit)/total time with available MAP data]*100

^**§**^ T0 was the time-point, when vasopressor support was initiated

^**ϕ**^ New significant AKI was defined as a peak shift of at least two AKI stage as per KDIGO criteria within 14 days after T0

^**δ**^ Major adverse kidney event (MAKE)−14 was a composite outcome of death, or new renal replacement therapy during the first 14 days, or doubling of serum creatinine from pre-illness level on day 14 or on day of discharge from ICU, whichever was earlier

^**γ**^ % Peak increase in serum creatinine = [(Peak creatinine value during 14 days after T0—creatinine level at randomization)/creatinine level at randomization]*100, among patients who did not receive renal replacement therapy

### Clinical efficacy and safety events

The peak percentage increase in serum creatinine was 57% [IQR: 33–121] versus 45% [14–61], *p* = 0.54, in the standard MAP versus individualized MAP group (Table 2). The trends for daily peak creatinine values over time were similar for both groups (Figure S5). Other renal outcomes including MAKE-14, new significant AKI, or need for RRT were similar for both groups. Day 14 mortality in the standard MAP group was 3 out of 20 (15%) and in the individualized MAP group was 1 out of 17 (6%), *p* = 0.61. There were no significant differences in the distribution of cardiovascular adverse events between the two groups (Table 2).

## Discussion

In this multicenter pilot RCT among vasopressor-treated patients with shock, an individualized MAP target strategy was demonstrated to be feasible. Although this study was terminated early, the pre-specified effect size in relation to the absolute difference in percentage MAP deficit between the two groups was achieved. Compared to the standard arm, the intervention was effective in achieving an adequate degree of separation over multiple days in the time-weighted average MAP deficit and percentage time with > 20% MAP deficit. Further, no significant between-group differences in any of the preliminary efficacy outcomes or adverse events were observed.

In the standard MAP group, the key exposure variables of time-weighted average MAP deficit and the proportion of time spent with > 20% MAP deficit, and the preliminary efficacy outcomes of MAKE-14 and 14-day mortality, were similar to those reported in the previous observational studies [9, 16]. Data from prior studies constituting REACT Shock study program [9, 16, 20–22] have consistently indicated the need to further investigate merits of individualized MAP targets in a high-quality definitive RCT.

Accordingly, we have now set out to conduct an investigator-initiated, parallel-group, multicenter, international, RCT (the REACT Shock RCT) among 1260 participants, to test whether a strategy of individualized MAP targets, based on patients’ usual pre-illness blood pressure measurements, during vasopressor therapy for shock in ICU, can improve 14-day mortality and MAKE-14. This pivotal trial will provide evidence to fulfill a crucial knowledge gap regarding a common and a fundamental intervention in critical care. Based on the effect size observed in our prospective cohort study [9], before and after study [16], and this pilot RCT, a sample size of 1260 will be required to demonstrate an absolute risk reduction of 6% in the primary endpoint (14-day mortality) with the intervention of individualized MAP target, assuming a 20% incidence of 14-day mortality in the standard care arm, at an alpha level of 0.05 and power of 80%, and allowing for 2.5% attrition. We welcome expressions of interest from investigators or centers who may wish to collaborate or participate in this next phase of research.

In addition to providing preliminary data on feasibility and clinical efficacy of this intervention, it informs the study workflow and procedures for the definitive RCT. Strengths of this study are that it was conducted at two centers in two countries imparting a degree of external validity. The design was pragmatic, and the study endpoints were pre-specified. Consistent with previous RCTs that also investigated effects of vasopressors in both septic and cardiogenic shock in the same setting [23, 24], we included both subgroups of septic and cardiogenic shock. Maintaining an acceptable level of blood pressure is the core principle of management for circulatory shock [25], regardless of whether it is a distributive state or a vasoconstrictive state. Vasopressor therapy is generally aimed at targeting a standard mean arterial blood pressure threshold of about 65 mmHg for both septic shock and cardiogenic shock [3, 5, 26, 27]. Since blood pressure targets are not different for these two shock states, we did not have any a priori reason to believe that physiological effects of a relative deficit in blood pressure would be much different for these two shock states.

Cardiovascular adverse events, including mortality, were not dissimilar between the two groups. Although there are several reports showing association between vasopressor dose and poor clinical outcomes, we are unaware of any clear evidence that vasopressor dose, when specifically titrated to minimize relative hypotension, is associated with adverse outcome. In the SEPSISPAM RCT [6], patients randomized to higher MAP target arm were indiscriminately treated with higher doses of norepinephrine regardless of their pre-illness blood pressure levels. The INPRESS RCT [28], among patients undergoing abdominal surgery, tested a similar strategy, where systolic blood pressure target was adjusted to preoperative blood pressure level, against standard management. Although patients in the intervention arm were exposed to higher vasopressor dose, the intervention reduced the risk of postoperative organ dysfunction without any significant increase in adverse events [28].

There are some limitations to note. It was an open label study. The study was not powered to detect statistically significant differences in clinical endpoints, consistent with its pilot design. As this study was recruiting during the COVID-19 pandemic, there were some delays in recruitment process which were beyond our control. Data related to other co-interventions after randomization such as fluid management, sedation interruption, ventilator weaning, nutrition, and use of steroids are not available. Data were recorded in the ICU observations charts as microgram/kg/min of the norepinephrine base, which avoided the issue of possibly different drug concentrations in use in different sites. The lack of medical equipoise was the foremost reason for exclusion and it covered conditions, where it might be relatively contraindicated to aim for a specifically lower or higher MAP target. As a pragmatic trial, it was difficult to list all the conditions under exclusion criteria where a particular blood pressure target may not be clinically suitable. Therefore, it was left to the clinicians near bedside to determine eligibility for enrollment. The other three major reasons were limitations of care, post-trauma/bleeding, or the need for dialysis. This perhaps reflects that patients with shock in an ICU can come with a vast range of diagnoses and certain blood pressure targets may not be clinically suitable in certain conditions.

Of note, the achieved MAP in the standard arm was higher than 65 mmHg. This is perhaps a reflection of standard practice, where in an attempt to maintain MAP at or above the minimum threshold of 65 mmHg, there is often an overshoot, and achieved MAP, while on vasopressors, is generally in the range of 70–80 mmHg. This is consistent with data on achieved MAP reported in other major RCTs such as ADRENAL RCT [29] and the usual care group of the JAMA-65 trial [30].

The mortality rate was somewhat lower in this pilot-RCT and this is perhaps a reflection of the strict inclusion and exclusion criteria resulting in a narrower cohort of patients. The day 14 mortality in the standard care arm was similar as that of our previous observational study [9] in a similar cohort. It is also possible that given the median time from ICU admission or vasopressor start to randomization in this study, some patients may be in the resolution phase of their shock state at the time of randomization. Lastly, the baseline imbalance in the distribution of cardiogenic shock and lactate was perhaps a manifestation of the modest size of this pilot RCT.

## Conclusions

In summary, this pilot RCT demonstrates that an individualized MAP target strategy is feasible and reasonably safe to implement. The design and conduct of this pilot trial have directly informed the protocol of a multicenter, phase III randomized controlled trial (REACT Shock RCT), which is currently underway.

## Supplementary information

Supplementary material 1

## Data Availability

Data can be made available to prospective researchers on a reasonable request to the corresponding author.
