# Supplementary material for: A pilot multicenter randomized controlled trial on individualized blood pressure targets versus standard care among critically ill patients with shock
Source: J Intensive Care. 2025 May 27;13:29. doi: 10.1186/s40560-025-00798-8 (PMC12107948; doi:10.1186/s40560-025-00798-8)
Supplement: Supplementary file 1 — Supplementary material 1 [file 40560_2025_798_MOESM1_ESM.docx]

**Online supplement**

**A pilot multicenter randomized controlled trial on individualized blood pressure targets versus standard care among critically ill patients with shock**

Rakshit Panwar *et al.*

**Supplementary Figures**

**Figure S1: Percentage MAP-deficit over time in standard MAP target group versus Individualized MAP target group for each participant with a loess smoother and 95% confidence interval summarising the trend for each group**

**
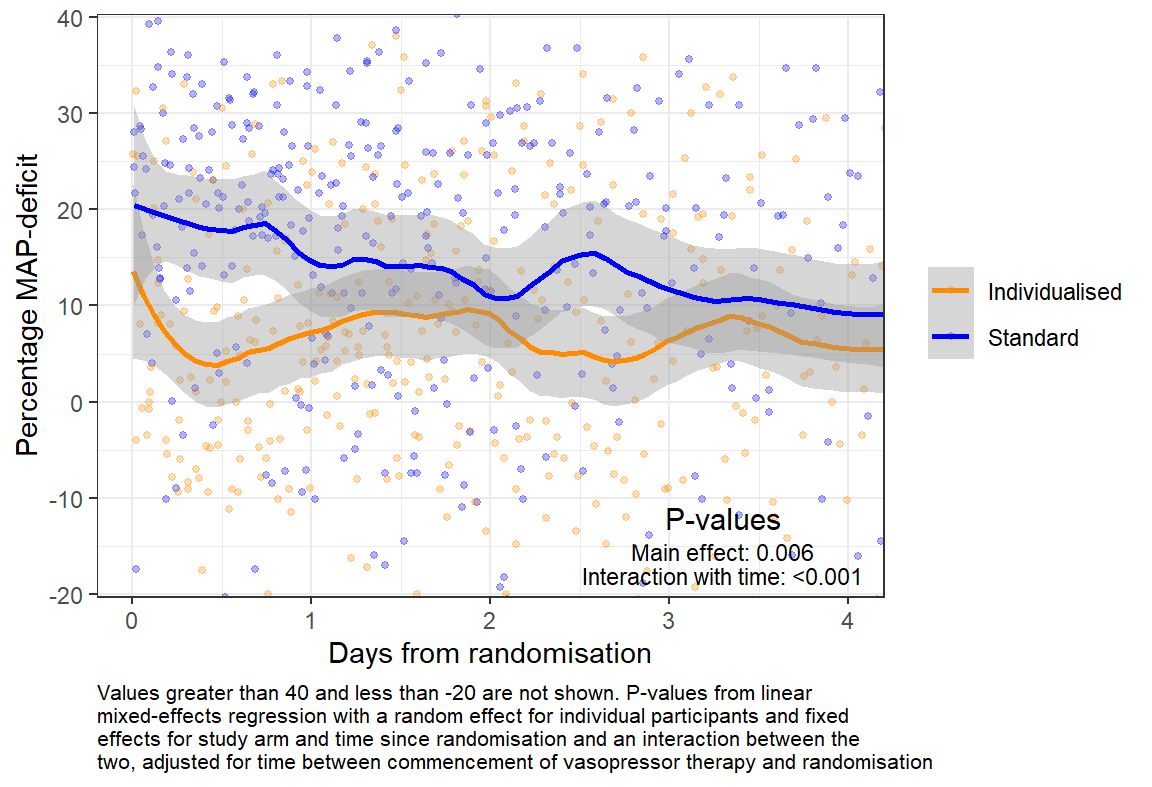
**

**Figure S2: Achieved MAP in ICU over time in standard MAP target group versus Individualized MAP target group for each participant with a loess smoother and 95% confidence interval summarising the trend for each group**

**
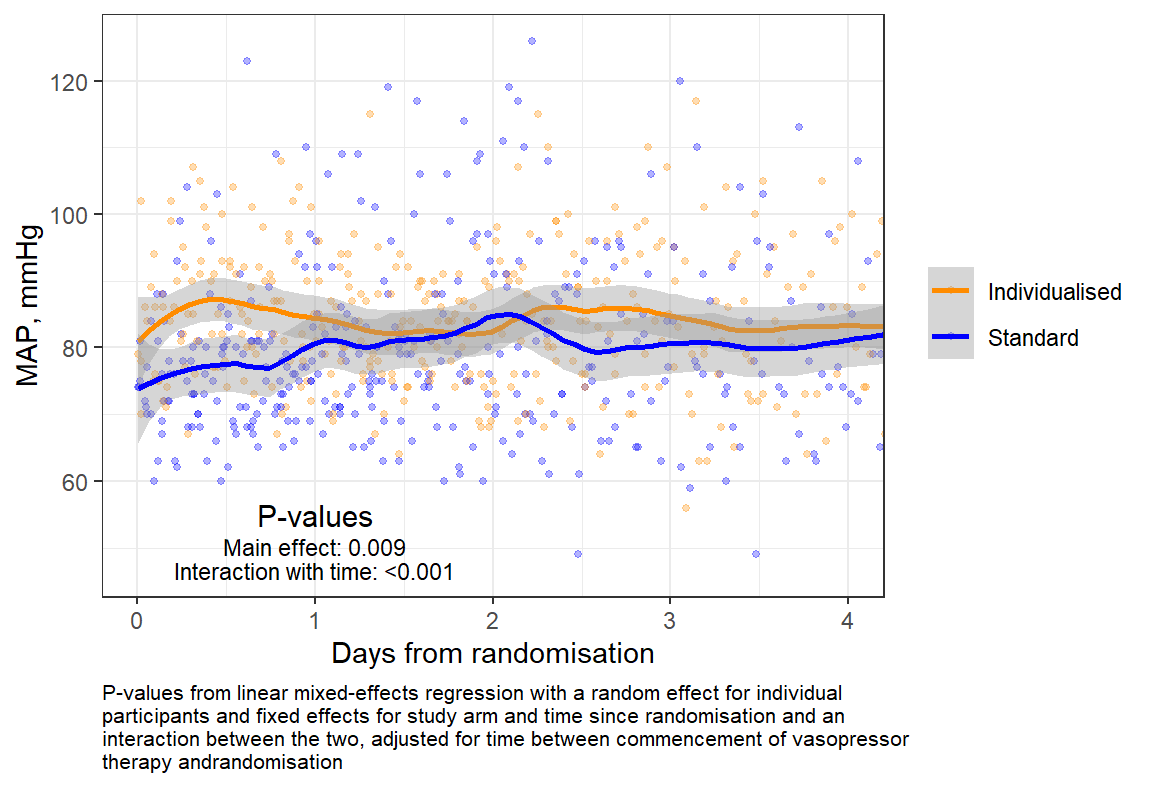
**

**Figure S3: Highest MAP each day (mean and 95% confidence intervals) in standard MAP target group versus Individualized MAP target group**

**
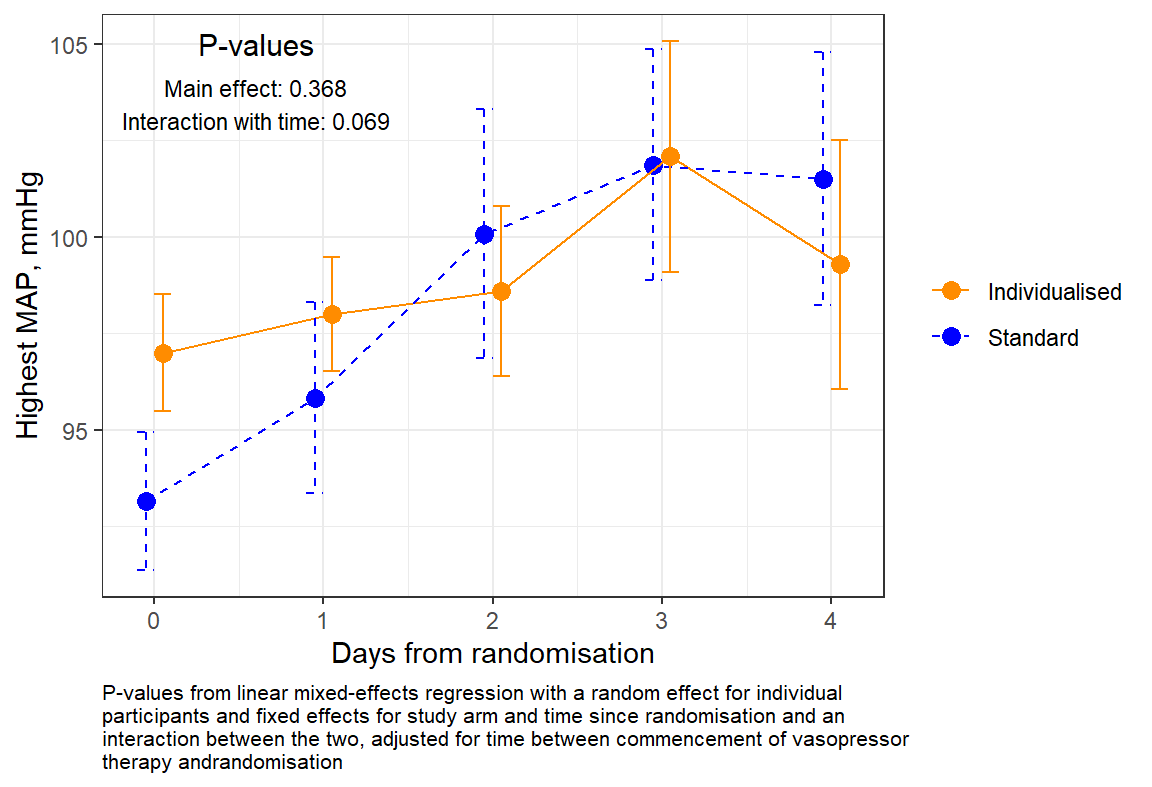
**

**Figure S4: Lowest MAP each day (mean and 95% confidence intervals) in standard MAP target group versus Individualized MAP target group**

**
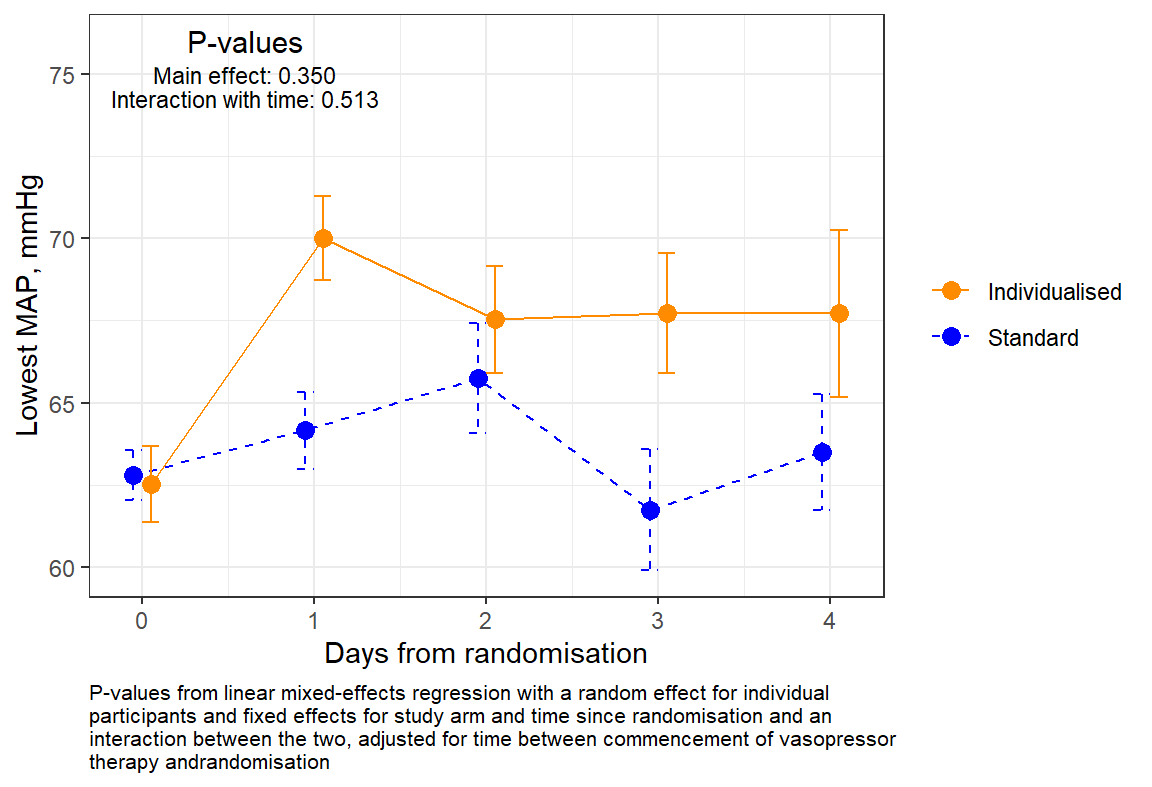
**

**Figure S5: Daily peak creatinine (mean and 95% confidence intervals) in standard MAP target group versus Individualized MAP target group**

**
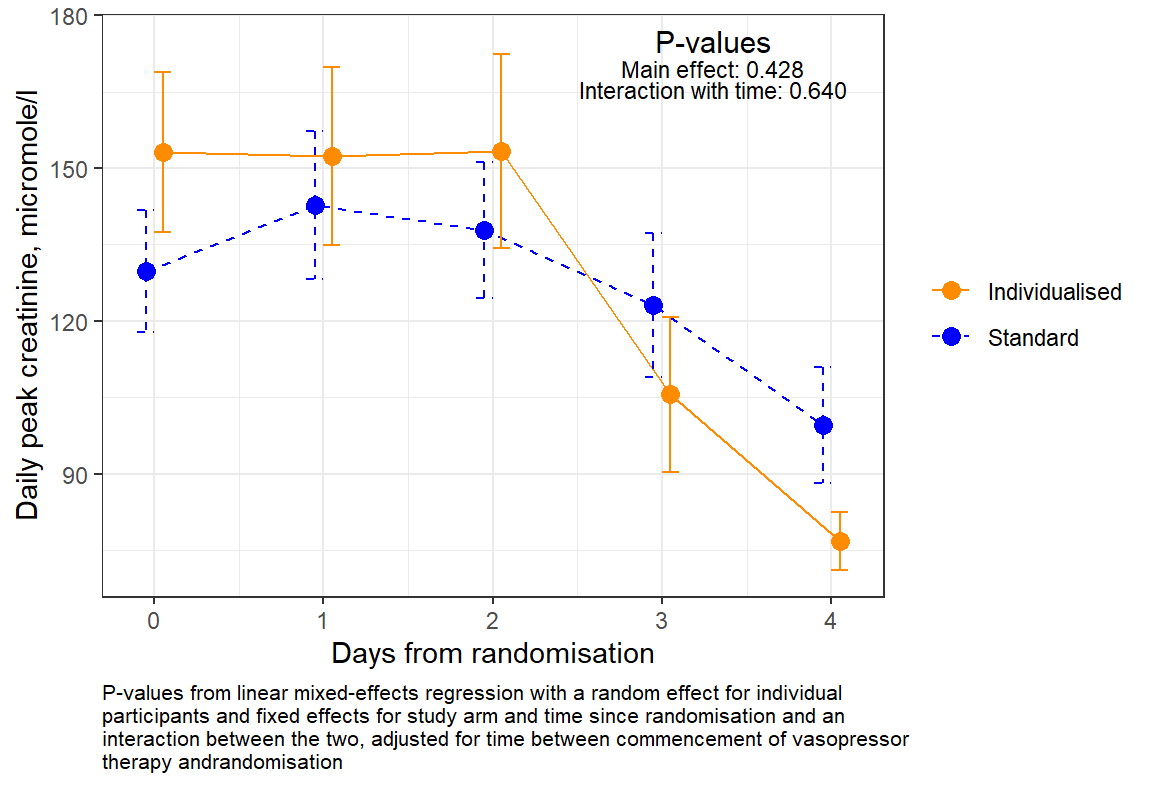
**

**Explanation of the primary endpoint**

The ‘time-weighted average MAP-deficit’ was a summary measure of percentage MAP-deficit to which patients were exposed from the time of randomisation to the last recorded MAP during vasopressor therapy in ICU. It was used to quantify the magnitude of average relative hypotension load for each patient (as done previously).^1^ At each four hourly time-interval during the study period, the percentage MAP-deficit was derived using the equation [% MAP-deficit = 100*[(pre-illness MAP−achieved MAP) /pre-illness MAP], where ‘pre-illness MAP’ was derived as a mean of patient’s most recent pre-illness blood pressure readings (as explained in the methods) and ‘achieved MAP’ was the MAP recorded at punctual timepoints on the ICU observation charts. The time-weighted average value for percentage MAP-deficit was then derived as an aggregate area-under-the-curve for % MAP-deficit divided by the cumulative time exposure to vasopressor for each individual patient.^1,2^ An illustration of this concept using hypothetical values is presented below.

**CONSORT 2016 Extension Checklist: Pilot and Feasibility Randomised Trials^3^**

| Item | Checklist Description | Response |
| --- | --- | --- |
| 1a | Identification as a pilot or feasibility randomized trial in the title | Yes – the title clearly identifies this as a pilot randomized trial. |
| 1b | Structured summary of pilot trial design, methods, results, and conclusions | Yes – the abstract is structured and includes design, interventions, key feasibility outcomes, and interpretation. |
| 2a | Scientific background and explanation of rationale for future definitive trial and reasons for randomized pilot trial | Yes – the background explains the need for individualized MAP targets and the rationale for testing feasibility before a full trial. |
| 2b | Specific objectives or research questions for pilot trial | Yes – the main feasibility objectives were to assess overall MAP-deficit and the percentage of timepoints with >20% MAP-deficit. |
| 3a | Description of pilot trial design (such as parallel, factorial) including allocation ratio | Yes – parallel group design with 1:1 allocation ratio. |
| 3b | Important changes to methods after pilot trial commencement (such as eligibility criteria), with reasons | Yes – see Supplementary Table S1 describing a protocol amendment to eligibility criteria during the trial. |
| 4a | Eligibility criteria for participants | Yes – inclusion and exclusion criteria clearly described in the Methods section. |
| 4b | Settings and locations where data were collected | Yes – conducted at two tertiary-level ICUs in Australia and Ireland. |
| 4c | How participants were identified and consented | Yes – details included in Methods. Patients were screened by research staff and consent was obtained from patients or surrogates. |
| 5 | The interventions for each group with sufficient details to allow replication | Yes – detailed description of how individualized MAP targets were determined, and standard MAP management provided. |
| 6a | Completely defined prespecified assessments or measurements to address each pilot trial objective | Yes – feasibility assessed using MAP-deficit and time spent >20% MAP-deficit. |
| 6b | Any changes to pilot trial assessments or measurements after trial commenced | Yes – see Supplementary Table S1 detailing protocol amendment relevant to eligibility criteria and time windows. |
| 7a | How sample size was determined | Sample size based on pragmatic estimate of patients required to evaluate feasibility measures. |
| 7b | When applicable, explanation of any interim analyses and stopping guidelines | No formal interim analysis; trial was stopped to enable the initiation of the phase III RCT. |
| 8a | Method used to generate the random allocation sequence | Yes – computer-generated random sequence. |
| 8b | Type of randomization; details of any restriction (such as blocking and block size) | Yes – stratified block randomization with variable block sizes. |
| 9 | Mechanism used to implement the random allocation sequence (e.g. sequentially numbered containers), describing any steps taken to conceal the sequence until interventions were assigned | Yes – allocation was implemented using sealed opaque envelopes by site investigators. |
| 10 | Who generated the random allocation sequence, who enrolled participants, and who assigned participants to interventions | Yes – randomization was generated by a third-party not involved in trial conduct. |
| 11a | Blinding (masking) after assignment to interventions | No – open-label pragmatic trial; blinding not feasible. |
| 11b | If relevant, description of similarity of interventions | Not applicable – intervention involved individualized MAP targets. |
| 12 | Methods used to address each pilot trial objective | Yes – MAP-deficit was calculated, and linear mixed models and summary statistics were used. |
| 13a | Participant flow (a diagram is strongly recommended) | Yes – Figure 1 provides CONSORT-style flow diagram. |
| 13b | Losses and exclusions after randomization, with reasons | Yes – reported in flow diagram and text. |
| 14a | Dates defining the periods of recruitment and follow-up | Yes – clearly specified in the Results section. |
| 14b | Why the pilot trial ended or was stopped | Trial was stopped upon funding approval for the full RCT to avoid delay. |
| 15 | A table showing baseline demographic and clinical characteristics for each group | Yes – Table 1 in the manuscript. |
| 16 | For each objective, number of participants included in each analysis and whether the analysis was by original assigned groups | Yes – reported in Results, with all analyses by assigned groups. |
| 17 | For each objective, results including expressions of uncertainty (such as 95% confidence interval) | Yes – medians, IQRs, or 95% CIs reported for key outcomes. |
| 18 | Results of any other analyses performed that could be used to inform the future definitive trial | Yes – includes MAP separation, dose metrics, and safety data. |
| 19 | Harms | Yes – adverse events and safety outcomes included. |
| 20 | Comment on pilot trial limitations | Yes – limitations section addresses real-world design, sample size, and MAP target variability. |
| 21 | Generalizability (external validity, applicability) of pilot trial methods and findings | Yes – generalizability discussed in relation to pragmatic design and settings. |
| 22 | Interpretation consistent with pilot trial objectives and findings, balancing potential benefits and harms, and considering other relevant evidence | Yes – clearly stated that this was a feasibility trial; interpretation is exploratory. |
| 23 | Implications for progression to a definitive trial, including any proposed amendments | Yes – findings informed protocol for REACT Shock RCT. No formal progression criteria set; funding and timing guided transition. |
| 24 | Registration number and name of trial registry | Yes – ACTRN12618000571279. |
| 25 | Where the pilot trial protocol can be accessed, if available | Yes – protocol provided as supplementary file. |
| 26 | Sources of funding and other support; role of funders | Yes – disclosed in Funding section. |

**Table S1: Protocol variations**

|  | **Item** | **Change** | **Reason for change** |
| --- | --- | --- | --- |
| 1. | Data management | Data management center was changed to Hunter Medical Research Institute from the George Institute of global health. | The study RedCap database was moved to the HMRI. |
| 2. | Secondary objectives | Secondary objective of rate of enrolment was replaced by time to death through day 90. | COVID-19 pandemic-related restrictions to resource allocation hindered the recruitment process making it difficult to extrapolate enrolment rate to the main phase 3 RCT. |
| 3. | Inclusion criteria | Removed the criteria of being within 48 hours of ICU admission and the need for positive pressure ventilation. Added two more supporting criteria for shock:   - Respiratory rate >22 per minute - Altered mentation | The 48-hour window criterion was removed since some patients could develop shock after few days of stay in ICU.  Some patients with shock might not be receiving respiratory support. Added two more surrogate markers of shock as per the qSOFA score. |
| 4. | Exclusion criteria | Added exclusion criteria of 24 hours or more being lapsed after initiation of vasopressor or inotropic support | To reduce mixed exposure to degree of MAP deficit, it was deemed important to exclude patients who have already spent more than 24 hours on vasopressor or inotropic support. |
| 5. | Study interventions | Need for mean perfusion pressure measurements was removed. Intervention aimed to minimize MAP deficit only. | This was simplified to improve compliance. |
| 6. | Cessation of study intervention | Study intervention to be ceased if a patient was considered well enough by the treating clinician for discharge out of ICU. | Decision about cessation of study intervention was simplified and kept at the discretion of the treating clinician. |
| 7. | Control group | Vasopressor support to be titrated to maintain a default MAP of 65 mmHg, unless a different MAP is specified by the treating team. | This was added for more clarity as current guidelines strongly recommend a target MAP of 65 mmHg for critically ill patients with shock. |
| 8. | Cardiovascular adverse events | Cardiovascular adverse events such as new-onset atrial or ventricular arrhythmia or cardiac arrest requiring chest compressions or defibrillation, mesenteric or myocardial ischemia, or bilateral digital ischemia during the study period to be monitored for all patients. | These were added as safety measures so that they can be compared between both arms. |

**Table S2: Vasopressor dose when given (median and interquartile range)**

| **Individual vasopressor use on each day** | **Individualized MAP group** | **Standard MAP group** |
| --- | --- | --- |
| **Noradrenaline (mcg/kg/min)** | | |
| Day 0 | 0.141 (0.103 - 0.25) | 0.12 (0.034 - 0.155) |
| Day 1 | 0.088 (0.053 - 0.18) | 0.068 (0.02 - 0.107) |
| Day 2 | 0.083 (0.028 - 0.127) | 0.032 (0.012 - 0.04) |
| Day 3 | 0.073 (0.022 - 0.108) | 0.05 (0.045 - 0.052) |
| Day 4 | 0.043 (0.023 - 0.129) | 0.064 (0.042 - 0.092) |
| **Adrenaline (mcg/kg/min)** | | |
| Day 0 | 0.122 (0.081 - 0.162) | - |
| Day 1 | 0.111 (0.064 - 0.158) | - |
| Day 2 | 0.068 (0.039 - 0.096) | - |
| **Vasopressin (units/min)** | | |
| Day 0 | 0.033 (0.025 - 0.04) | 0.032 (0.017 - 0.04) |
| Day 1 | 0.019 (0.015 - 0.03) | 0.033 (0.02 - 0.037) |
| Day 2 | 0.028 (0.021 - 0.034) | 0.026 (0.018 - 0.033) |
| Day 3 | 0.028 (0.028 - 0.028) | 0.022 (0.022 - 0.022) |
| **Dobutamine (mcg/kg/min)** | | |
| Day 0 | 2.66 (2.66 - 2.66) | - |
| Day 1 | 0.443 (0.443 - 0.443) | - |

**References**

1. Panwar R, Tarvade S, Lanyon N, et al. Relative Hypotension and Adverse Kidney-related Outcomes among Critically Ill Patients with Shock. A Multicenter, Prospective Cohort Study. *Am J Respir Crit Care Med*. 11 15 2020;202(10):1407-1418. doi:10.1164/rccm.201912-2316OC

2. Panwar R, Van Haren F, Cazzola F, Nourse M, Brinkerhoff G, Quail A. Standard care versus individualized blood pressure targets among critically ill patients with shock: A multicenter feasibility and preliminary efficacy study. *J Crit Care*. May 04 2022;70:154052. doi:10.1016/j.jcrc.2022.154052

3. Eldridge SM, Chan CL, Campbell MJ, et al. CONSORT 2010 statement: extension to randomised pilot and feasibility trials. *BMJ*. Oct 24 2016;355:i5239. doi:10.1136/bmj.i5239
